# TRAITER: transformer-guided diagnosis and prognosis of heart failure using cell nuclear morphology and DNA damage marker

**DOI:** 10.1093/bioinformatics/btae610

**Published:** 2024-10-16

**Authors:** Hiromu Hayashi, Toshiyuki Ko, Zhehao Dai, Kanna Fujita, Seitaro Nomura, Hiroki Kiyoshima, Shinya Ishihara, Momoko Hamano, Issei Komuro, Yoshihiro Yamanishi

**Affiliations:** Department of Bioscience and Bioinformatics, Faculty of Computer Science and Systems Engineering, Kyushu Institute of Technology, Iizuka 820-8502, Fukuoka, Japan; Department of Cardiovascular Medicine, Graduate School of Medicine, The University of Tokyo, Bunkyo, Tokyo 113-8655, Japan; Department of Frontier Cardiovascular Science, Graduate School of Medicine, The University of Tokyo, Bunkyo, Tokyo 113-8655, Japan; Department of Cardiovascular Medicine, Graduate School of Medicine, The University of Tokyo, Bunkyo, Tokyo 113-8655, Japan; Department of Cardiovascular Medicine, Graduate School of Medicine, The University of Tokyo, Bunkyo, Tokyo 113-8655, Japan; Department of Computational Diagnostic Radiology and Preventive Medicine, Graduate School of Medicine, The University of Tokyo, Bunkyo, Tokyo 113-8655, Japan; Department of Cardiovascular Medicine, Graduate School of Medicine, The University of Tokyo, Bunkyo, Tokyo 113-8655, Japan; Department of Frontier Cardiovascular Science, Graduate School of Medicine, The University of Tokyo, Bunkyo, Tokyo 113-8655, Japan; Department of Bioscience and Bioinformatics, Faculty of Computer Science and Systems Engineering, Kyushu Institute of Technology, Iizuka 820-8502, Fukuoka, Japan; Department of Bioscience and Bioinformatics, Faculty of Computer Science and Systems Engineering, Kyushu Institute of Technology, Iizuka 820-8502, Fukuoka, Japan; Department of Bioscience and Bioinformatics, Faculty of Computer Science and Systems Engineering, Kyushu Institute of Technology, Iizuka 820-8502, Fukuoka, Japan; Department of Cardiovascular Medicine, Graduate School of Medicine, The University of Tokyo, Bunkyo, Tokyo 113-8655, Japan; Department of Frontier Cardiovascular Science, Graduate School of Medicine, The University of Tokyo, Bunkyo, Tokyo 113-8655, Japan; International University of Health and Welafare, Minato, Tokyo 107-8402, Japan; Department of Bioscience and Bioinformatics, Faculty of Computer Science and Systems Engineering, Kyushu Institute of Technology, Iizuka 820-8502, Fukuoka, Japan; Department of Complex Systems Science, Graduate School of Informatics, Nagoya University, Chikusa, Nagoya, Aichi 464-8601, Japan

## Abstract

**Motivation:**

Heart failure (HF), a major cause of morbidity and mortality, necessitates precise diagnostic and prognostic methods.

**Results:**

This study presents a novel deep learning approach, Transformer-based Analysis of Images of Tissue for Effective Remedy (TRAITER), for HF diagnosis and prognosis. Using image segmentation techniques and a Vision Transformer, TRAITER predicts HF likelihood from cardiac tissue cell nuclear morphology images and the potential for left ventricular reverse remodeling (LVRR) from dual-stained images with cell nuclei and DNA damage markers. In HF prediction using 31 158 images from 9 patients, TRAITER achieved 83.1% accuracy. For LVRR prediction with 231 840 images from 46 patients, TRAITER attained 84.2% accuracy for individual images and 92.9% for individual patients. TRAITER outperformed other neural network models in terms of receiver operating characteristics, and precision–recall curves. Our method promises to advance personalized HF medicine decision-making.

**Availability and implementation:**

The source code and data are available at the following link: https://github.com/HamanoLaboratory/predict-of-HF-and-LVRR.

## 1 Introduction

Currently, the cumulative number of heart failure (HF) patients worldwide has surpassed 60 million (Dickstein *et al.* 2008, Travessa and De Menezes Falcão 2016, Vos *et al.* 2017), making it a persistent cause of morbidity and mortality (Braunwald 2015, Savarese and Lund 2017) despite advancements in cardiovascular treatment. With the aging global population, the incidence of HF is expected to rise (Okura *et al.* 2008, Benjamin *et al.* 2019). Dilated cardiomyopathy (DCM) is a leading cause of heart failure, characterized by left ventricular dilatation and impaired contractile function. Given the diverse causes and pathologies associated with HF, reliable methods for predicting disease status and post-treatment prognosis are crucial.

Prognosis in HF is highly heterogeneous among different patients. In patients with HF, the left ventricle undergoes remodeling in response to volume or pressure overload. Prolonged remodeling eventually results in dilation and reduced contractility of the left ventricle, which exacerbates HF (Schwarz *et al.* 1983, Merlo *et al.* 2011). Therefore, HF treatments aim to reverse the left ventricular dilation and restore contractility, which is known as left ventricular reverse remodeling (LVRR) (Matsumura *et al.* 2013, Merlo *et al.* 2015, Litjens *et al.* 2017). Achievement of LVRR in response to HF treatments is associated with a better prognosis. Previous studies have demonstrated that HF is characterized by an altered nuclear morphology (Ross and Stroud 2021) and activation of DNA damage (Ko *et al.* 2019, Dai *et al.* 2024), suggesting that nuclear morphology and DNA damage markers in cardiac tissue may serve diagnostic and prognostic purposes in HF.

Recently, deep learning models have demonstrated success in analyzing medical pathology images (Litjens *et al.* 2017), predicting brain age from magnetic resonance imaging images (Jonsson *et al.* 2019), cellular aging from nuclear morphology images (Heckenbach *et al.* 2022), and endometrial cancer recurrence from multiple immunofluorescence-stained images (Jiménez-Sánchez *et al.* 2023). These models offer a valuable contribution to precision medicine by introducing new evaluation metrics and enhancing the precise diagnosis of conditions that may be challenging for human observation. In the context of HF treatment, there is a strong motivation to develop reliable deep-learning models to support accurate clinical diagnosis and treatment decision-making, reducing dependence on manual assessment.

This study presents a novel deep learning-based approach, Transformer-based Analysis of Images of Tissue for Effective Remedy (TRAITER), designed for the diagnosis and prediction of the prognosis of HF. Prediction is executed through a series of image segmentation techniques and a Vision Transformer (ViT) (Dosovitskiy *et al.* 2021) based on pathological images of nuclei and DNA damage marker. TRAITER can predict the likelihood of HF from high-resolution nuclear morphology images and the probability of LVRR from dual-stained images of both nuclei and DNA damage marker. Notably, our approach is designed to be applicable even with the limited availability of endomyocardial biopsies from patients with HF. The study demonstrates the utility of the proposed method in diagnosing HF and predicting LVRR using stained images of nuclei and DNA damage marker from endomyocardial biopsies.

## 2 Materials and methods

### 2.1 Patient cohort for the HF prediction

This study was approved by the Ethics Committee of Kyushu Institute of Technology (21–07) and the University of Tokyo (number 11801). We included six patients diagnosed with DCM retrospectively, all of whom were hospitalized for HF and had undergone endomyocardial biopsy at the University of Tokyo Hospital between 2016 and 2020. The diagnosis of DCM was established through various modalities, including coronary angiography, echocardiography, and endomyocardial biopsy (McNamara *et al.* 2011, Japp *et al.* 2016). We incorporated three patients who had undergone endomyocardial biopsy within the same timeframe after heart transplantation, and these individuals, displaying no histopathological rejection, constituted the non-HF control group.

### 2.2 Patient cohort for the LVRR prediction

We conducted a retrospective enrollment of patients diagnosed with DCM who had undergone endomyocardial biopsy at the hospital between 2016 and 2020. To form the predictive cohort for LVRR, patients with unstable hemodynamics, those receiving mechanical support (such as intravenous catecholamine, intra-aortic balloon pumping, and percutaneous cardiopulmonary support) within 30 days preceding biopsy, were excluded. Furthermore, patients who had undergone left ventricular assist device implantation or heart transplantation before biopsy were excluded.

LVRR was defined as an increase ≥10% in the absolute value of left ventricular ejection fraction from the baseline measurement accompanying endomyocardial biopsy to a final value >35%. This definition was based on the echocardiography reevaluation 12 months post-endomyocardial biopsy. Patients experiencing cardiac death, undergoing heart transplantation, or receiving left ventricular assist device implantation within 12 months were classified as LVRR-negative.

### 2.3 Immunofluorescence staining in the HF prediction cohort

Four-μM-thick sections were generated from formalin-fixed, paraffin-embedded specimens obtained through endomyocardial biopsies of six patients experiencing HF due to DCM, along with three control subjects. To visualize nuclear morphology, tissue slides were stained using DAPI (1:1000; Dojindo Molecular Technologies, Inc., Kumamoto, Japan).

Using a Zeiss LSM880 inverted confocal microscope (Carl Zeiss Co., Ltd, Tokyo, Japan) with a 40× objective, high-resolution nuclear morphology images were acquired. The Z-stack and tile scan functions were used to capture images encompassing the entire area and thickness of a single specimen.

### 2.4 Immunofluorescence staining in the LVRR prediction cohort

Four-μM-thick sections were prepared from formalin-fixed, paraffin-embedded specimens obtained through endomyocardial biopsies of 23 subjects classified as LVRR-positive and 23 subjects classified as LVRR-negative. Staining for DNA damage marker involved the use of the anti-γ-H2A.X antibody (#9718, 1:200, Cell Signaling Technology, Danvers, MA, USA) with overnight incubation at 4°C, followed by incubation with a secondary antibody, anti-rabbit IgG-Alexa 647 (1:300, Thermo Fisher Scientific, Waltham, MA, USA), for 1 h at room temperature. Nuclei were counterstained with DAPI (1:1000; Dojindo Molecular Technologies, Inc., Kumamoto, Japan). Images were acquired using a Keyence BZ-X810 inverted fluorescence microscope at a 20× objective using two fluorescence channels, covering the entire area of a single specimen through the stitching function.

### 2.5 Segmentation of the cell nuclear morphology images of cardiac tissue for the HF prediction

To generate images depicting the nuclear morphology of cardiac tissue cells, an automated segmentation process was used to isolate each nucleus within all slide images. The segmentation process encompassed the following steps:

**Step 1.** The images were loaded into OpenCV (version 4.2.5.52), an open-source image processing library, and processed in grayscale using the Python (version 3.8.8) environment.

**Step 2.** To facilitate segmentation, the images were enlarged 16-fold using the Bicubic interpolation method. This step was necessary due to the small size of human cardiac tissue cellular nuclei in this study, which posed challenges for straightforward segmentation.

**Step 3.** Grayscale images were subjected to Otsu’s binarization algorithm (Otsu 1996), converting them into binary images where pixels were classified as white (1) or black (0). This algorithm, based on luminance value distribution, maximizes separation and converts the image into a binary format.

**Step 4.** A morphological gradient (Haralick *et al.* 1987) was applied to extract the contour of cardiac tissue cell nuclei. This involved calculating the difference between expanded and contracted images.

**Step 5.** The coordinates and areas of the outlined objects were determined. Objects with an area of 512 pixels or less were identified. Bounding rectangle coordinates were determined, allowing for the individual extraction of cardiac tissue cell nuclei based on these coordinates.

### 2.6 Image overlay for LVRR prediction

We overlayed the images stained for nuclei and DNA damage marker γ-H2A.X, which were simultaneously acquired, to create dual-stained images. Adjustments to the brightness and contrast of the double-stained images were made using ImageJ (Abràmoff *et al.* 2004). The Auto function was used to analyze the brightness and contrast histograms in each image, facilitating automatic optimization.

### 2.7 Image segmentation for LVRR prediction

Given that only one double-stained image was available per patient, we executed image segmentation to increase the number of images for deep learning. This involves extracting a portion of the original image and storing its distinct image patch. Multiple 128 × 128 pixel images were extracted, resulting in the generation of 5040 patches per patient.

### 2.8 Construction of a dataset of nuclear morphology images for HF prediction

An image dataset for HF prediction was curated from nuclear morphology images of cardiac tissue. The training and test sets were exclusive, ensuring that no images from the same patient appeared in both sets. The number of images within the training sets was augmented by rotating images in four directions.

### 2.9 Construction of a dataset of dual-stained images for LVRR prediction

An image dataset for LVRR prediction was curated from segmented patches of dual-stained images. The training and test sets were exclusive, ensuring that no images from the same patient appeared in both sets.

### 2.10 Pre-training and fine-tuning of the deep learning models

To overcome the constraint of a limited image pool for analysis, we conducted pre-training of deep learning models on the extensive ImageNet (Russakovsky *et al.* 2015) dataset (https://www.image-net.org). ImageNet comprises 14 197 122 color photographs with associated teacher labels, and it offers pre-trained models with optimized parameters (https://github.com/keras-team/keras/tree/v2.12.0/keras/applications). In our study, these pre-trained models from ImageNet were utilized, and fine-tuned on the images within the HF cohort and LVRR cohort data, leading to the creation of final predictive models tailored for HF and LVRR predictions.

### 2.11 Deep learning model used to predict HF

ResNet50 (He *et al.* 2016), InceptionResNetV2 (Szegedy *et al.* 2017), and Vision Transformer (ViT) (Dosovitskiy *et al.* 2021) were compared in our predictive framework. ResNet50 addresses the vanishing gradient problem by introducing a residual block that combines the output value of the convolutional layer with the input value. Comprising 50 blocks, including a convolutional layer with multiple residual blocks, ResNet50 was utilized. Additionally, Inception ResNetv2, which integrates an Inception module with residual connections, was adopted. Finally, ViT, applying a Transformer to the image field for computational efficiency, was implemented. The framework was developed using Python (version 3.11), Pytorch (version 1.11.0), and Timm (version 0.6.7).

### 2.12 Deep learning model used to predict LVRR

ResNet50 (He *et al.* 2016), InceptionResNetV2 (Szegedy *et al.* 2017), and ViT (Dosovitskiy *et al.* 2021) were compared for predicting LVRR. Two distinct approaches were applied for LVRR prediction: individual image prediction and individual patient prediction. In the case of individual image prediction, the model underwent training on all image patches within the training set, followed by predictions made on all image patches in the test set. Conversely, for individual patient prediction, the model was trained on all patient patches in the training set, and predictions were executed on all patient patches in the test set. The LVRR prediction label for each patient was determined through a majority vote based on the number of predicted patches derived from the same patient. Performance evaluation involved assessing the percentage of correct LVRR labels, comparing predictions with the actual diagnosis for each patient.

## 3 Results

### 3.1 Clinical characteristics and an experimental overview

**Figure 1.**
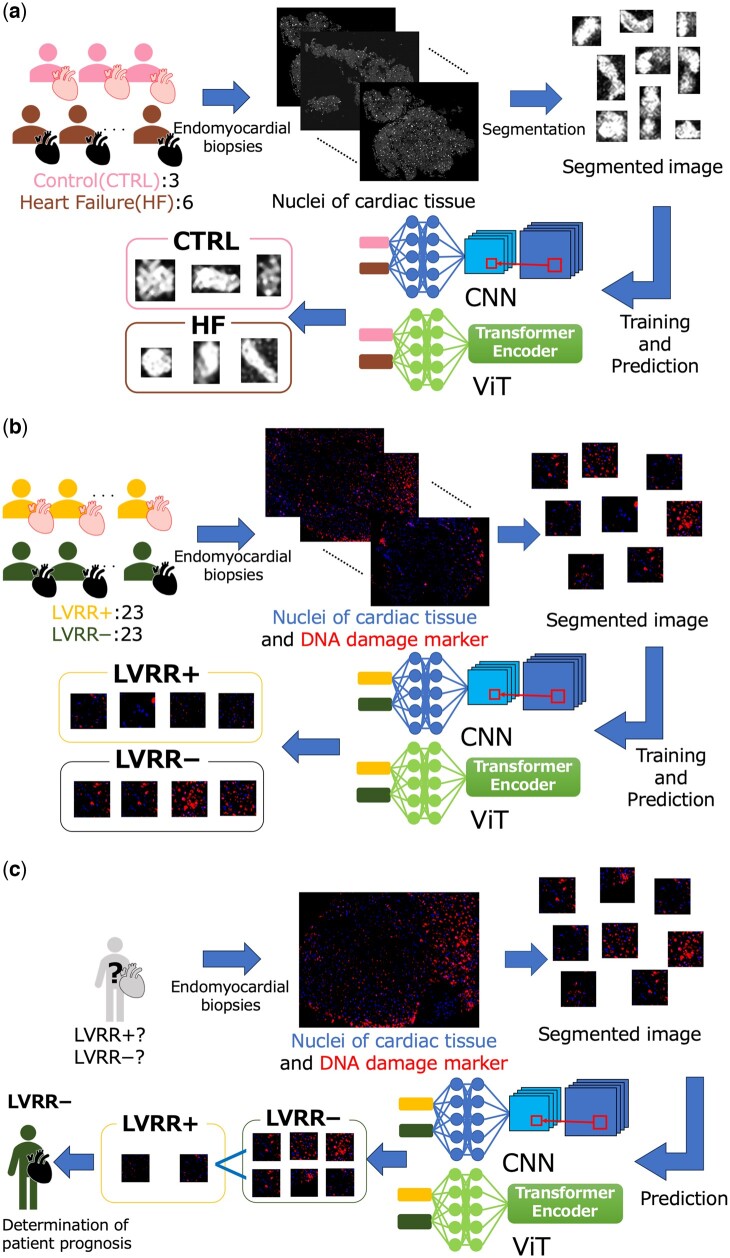
TRAITER overview for HF and LVRR prediction. (a) Overview of prediction of heart failure (HF) using nuclear morphology images of cardiac tissue. (b) Overview of prediction of left ventricular reverse remodeling (LVRR) for individual images derived from nuclei and DNA damage marker dual-stained images of cardiac tissue. (c) Overview of LVRR prediction for individual patients with HF based on nuclei and DNA damage marker dual-stained images.

In this study, we introduced a Vision Transformer-based method, referred to as TRAITER, aimed at predicting the likelihood of HF using high-resolution nuclear morphology images of cardiac tissue and predicting the likelihood of LVRR from dual-stained images with nuclei and DNA damage marker. Figure 1 provides an overview of HF prediction (Fig. 1a), LVRR prediction for individual images (Fig. 1b), and LVRR prediction for individual patients (Fig. 1c).

For HF prediction, we used medical images from six patients with dilated cardiomyopathy (DCM), which is a representative disease causing HF, who underwent endomyocardial biopsy at the University of Tokyo Hospital between 2016 and 2020. Furthermore, three patients who underwent endomyocardial biopsy during the same period after heart transplantation, displaying no histopathological rejection, were included as the non-HF control group. Endomyocardial biopsies were collected, and 4-μM-thick sections of formalin-fixed paraffin-embedded specimens were prepared, which were stained using 4’,6-diamidino-2-phenylindole (DAPI) for nuclei. A segmentation process isolated each nucleus in the whole-slide images, generating nuclear morphology images of cardiac tissue (Supplementary Fig. S1a). Representative nuclear morphology images of patients with HF and non-HF controls are shown in Supplementary Fig. S1b, and the detailed data statistics from nuclear morphology images are shown in Supplementary Fig. S1c. These images were then split into training and test sets to form a dataset for nuclear morphology images of cardiac tissue (Table 1). Images from the same patient were exclusively assigned to either the training or test sets. The training set was augmented by rotating images in four directions, resulting in 28 460 images, while the test set comprised 2698 images. Subsequently, a deep learning model was constructed using the nuclear morphology image dataset to predict the likelihood of HF in individual images.

**Table 1.** Data summary of the constructed image datasets for HF prediction.^a^

	Training		Test	
	Patients	Images	Patients	Images
CTRL	1	14 188	2	1346
HF	3	14 272	3	1352
Total	4	28 460	5	2698

a Dataset comprising nuclear morphology images of cardiac tissue. The number of patients and images are shown.

For LVRR prediction, we used medical images from 46 patients diagnosed with DCM who underwent endomyocardial biopsy at the University of Tokyo Hospital between 2016 and 2020. Endomyocardial biopsies were obtained, and 4-μM-thick sections of formalin-fixed paraffin-embedded specimens were prepared, which were stained using DAPI for nuclei and anti-γ-H2A.X antibody for DNA damage (Supplementary Fig. S2a). Overlaying these images produced a single dual-stained image (Supplementary Fig. S2b), which was then divided into smaller sections (patches) to create multiple patches (Supplementary Fig. S2c), forming a dual-stained image dataset for LVRR prediction (Table 2). The training set comprised 161 280 images, and the test set comprised 70 560 images. Importantly, images from the same patient were not split into training and test sets. Two types of predictions were made for LVRR: prediction for individual images and prediction for individual patients. The model for individual images was constructed based on a set of image patches, while the model for individual patients was constructed based on a set of patient patches. The LVRR prediction label for each patient was determined through a majority vote, considering the number of predicted patches from the same patient.

**Table 2.** Data summary of the constructed image datasets for LVRR prediction.^a^

	Training		Test	
	Patients	Images	Patients	Images
LVRR+	16	80 640	7	35 280
LVRR−	16	80 640	7	35 280
Total	32	161 280	14	70 560

a Dataset consisting of dual-stained images of nuclei and DNA damage marker. The number of patients and images are shown.

### 3.2 Performance evaluation of HF prediction

**Figure 2.**
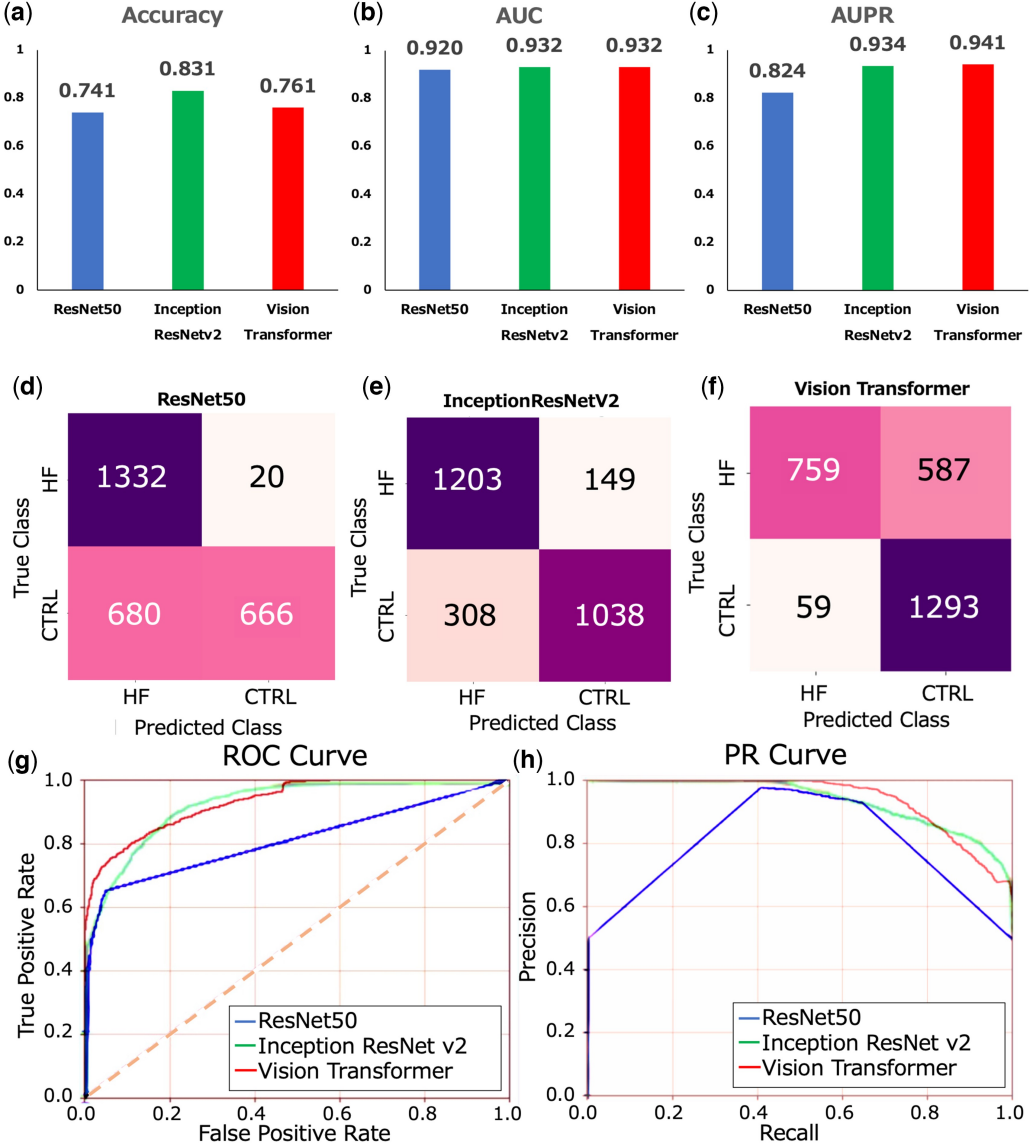
Performance evaluation of HF prediction from cell nuclear morphology images of cardiac tissue. (a) Model accuracies depicted by the blue bar for ResNet50, green bar for InceptionResNetv2, and red bar for Vision Transformer (ViT). (b) Area under the ROC curve (AUC) scores represented by the blue bar for ResNet50, green bar for InceptionResNetv2, and red bar for ViT. (c) Area under the precision–recall curve (AUPR) scores illustrated by the blue bar for ResNet50, green bar for InceptionResNetv2, and red bar for ViT. (d–f) Confusion matrices for ResNet50 (d), InceptionResNetv2 (e), and ViT (f). (g) Receiver operating characteristic (ROC) curves for ResNet50 (blue line), InceptionResNetv2 (green line), and ViT (red line). (h) Precision–recall (PR) curves for ResNet50 (blue line), InceptionResNetv2 (green line), and ViT (red line).

We applied our proposed TRAITER method to predict the likelihood of HF from nuclear morphology images of cardiac tissue. The Vision Transformer (ViT) model (Supplementary Fig. S3) within TRAITER underwent pre-training on ImageNet^.^ (Russakovsky *et al.* 2015) and fine-tuning on images within the HF cohort (a more detailed description of ViT can be found in the Supplementary Methods section). We evaluated the performance using a confusion matrix, receiver operating characteristic (ROC) curve, and precision–recall (PR) curve. Metrics such as accuracy, area under the ROC curve (AUC), and area under the PR curve (AUPR) were calculated (Fig. 2). ViT was compared with ResNet50 (He *et al.* 2016) and InceptionResNetv2 (Szegedy *et al.* 2017), widely used neural network models in image analysis research.

InceptionResNetv2 had the highest accuracy of 0.831 (Fig. 2a). ViT achieved the highest AUC score of 0.932 (Fig. 2b) and the highest AUPR score of 0.941 (Fig. 2c). The confusion matrix summarized true positives, false positives, true negatives, and false negatives for binary classification of HF or non-HF based on a prediction score threshold. Notably, InceptionResNetv2 exhibited high accuracy for the non-HF class, while ViT excelled in accuracy for the HF class (Fig. 2d–f). In terms of the ROC curve (Fig. 2g) and PR curve (Fig. 2h), ViT outperformed other neural network models, particularly in the high prediction score region, surpassing ResNet50 and InceptionResNetv2. However, in the low prediction score region, the curves for ViT dipped below those of ResNet50 and InceptionResNetv2. These findings demonstrate that ViT proves more beneficial for HF diagnosis when emphasizing high precision scores.

### 3.3 Performance evaluation on the LVRR prediction for individual images

We applied our proposed TRAITER method to predict the likelihood of LVRR using images of nuclei stained with DAPI and images of DNA damage marker stained with anti-γ-H2A.X, categorizing images into LVRR-positive or LVRR-negative classes. We evaluated the impact of dual-stained images, featuring nuclei and DNA damage marker, on prediction accuracy. Image segmentation was conducted for each stained image, leading to the creation of three image datasets for LVRR prediction: stained images of nuclei, stained images of DNA damage marker, and dual-stained images. The Vision Transformer model in TRAITER underwent pre-training on ImageNet (Russakovsky *et al.* 2015) and fine-tuning on images within the LVRR cohort.

We evaluated the performance through a confusion matrix, ROC curve, and PR curve, with calculations of accuracy, AUC, and AUPR scores (Supplementary Fig. S4). Using stained images of nuclei yielded an accuracy of 0.421, while stained images of DNA damage marker achieved a higher accuracy of 0.725, indicating an improvement in prediction accuracy. The use of dual-stained images significantly enhanced accuracy, achieved 0.842 (Supplementary Fig. S4a). Furthermore, the use of dual-stained images improved the classification of LVRR-positive and LVRR-negative images (Supplementary Figs S4d–f). Regarding AUC scores, stained images of nuclei achieved 0.373, stained images of DNA damage marker achieved 0.810, and dual-stained images achieved 0.924, affirming the superior performance of dual-stained images (Supplementary Fig. S4b). ROC curves corroborated the heightened performance of dual-stained images (Supplementary Fig. S4g). AUPR scores followed a similar tendency, with stained images of nuclei achieved 0.458, stained images of DNA damage marker achieved 0.807, and dual-stained images achieved 0.920, confirming the superior performance of dual-stained images (Supplementary Fig. S4c). PR curves further supported the enhanced performance of dual-stained images (Supplementary Fig. S4h). Therefore, dual-stained images were used for LVRR prediction below.

**Figure 3.**
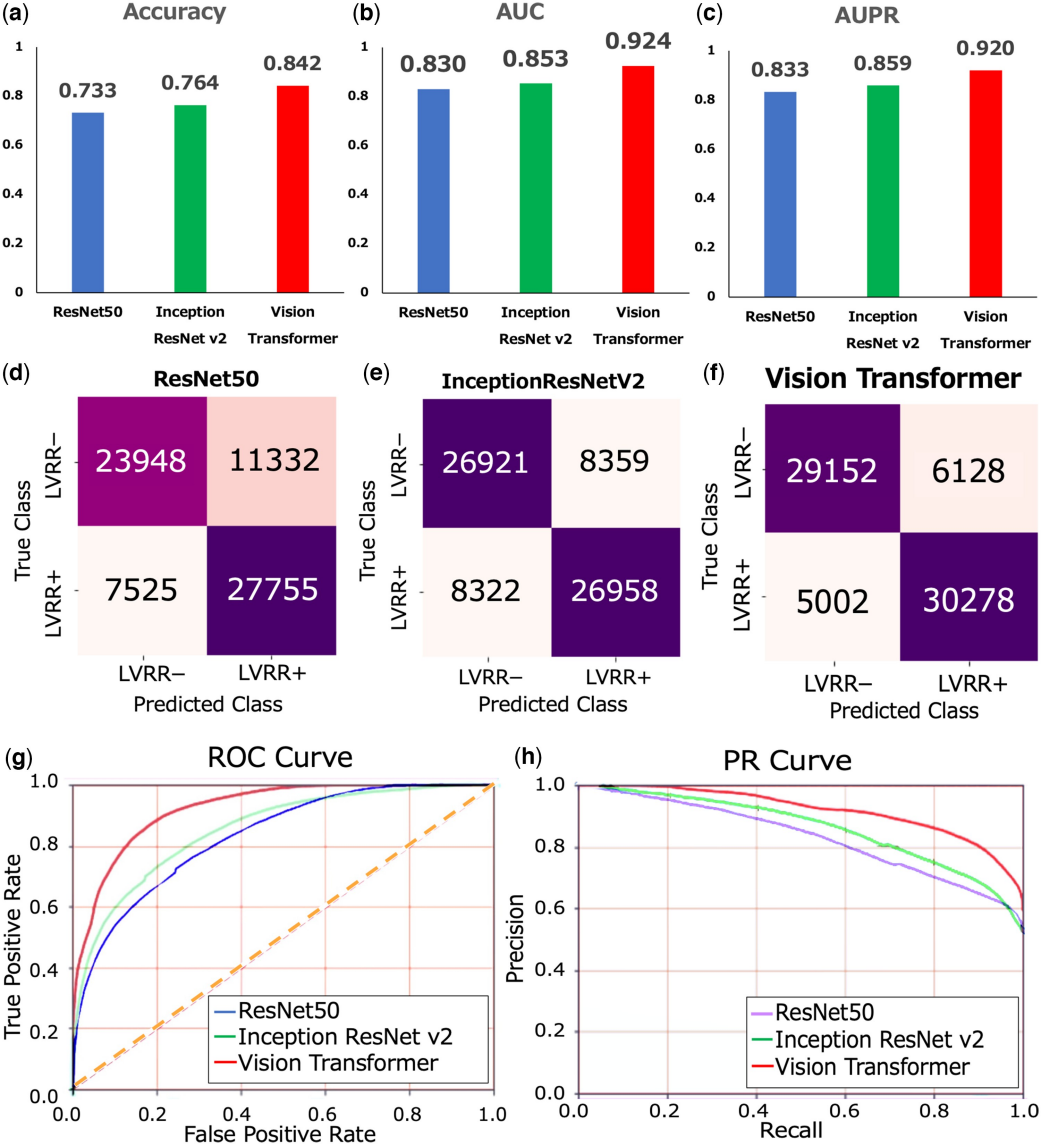
Performance evaluation of LVRR prediction for individual images from double-stained images of cell nuclei and DNA damage markers. (a) Model accuracies illustrated by the blue bar for ResNet50, green bar for InceptionResNetv2, and red bar for Vision Transformer (ViT). (b) AUC scores depicted by the blue bar for ResNet50, green bar for InceptionResNetv2, and red bar for ViT. (c) AUPR scores shown by the blue bar for ResNet50, green bar for InceptionResNetv2, and red bar for ViT. (d) Confusion matrix of ResNet50, (e) confusion matrix of InceptionResNetv2, and (f) confusion matrix of ViT. (g) ROC curves for ResNet50 (blue line), InceptionResNetv2 (green line), and ViT (red line). (h) PR curves for ResNet50 (blue line), InceptionResNetv2 (green line), and ViT (red line).

We compared the ViT model within TRAITER with ResNet50 (He *et al.* 2016) and InceptionResNetv2 (Szegedy *et al.* 2017) in the task of predicting LVRR. In contrast to ResNet50 (He *et al.* 2016) and InceptionResNetv2 (Szegedy *et al.* 2017) (Fig. 3), ViT achieved the highest accuracy at 0.842 (Fig. 3a). The confusion matrix further illustrated ViT’s accuracy in predicting both LVRR-positive and LVRR-negative images (Fig. 3d–f). Additionally, ViT obtained the highest AUC score of 0.924 (Fig. 3b) and the highest AUPR score of 0.920 (Fig. 3c). Comparative analysis of the ROC curve (Fig. 3g) and PR curve (Fig. 3h) revealed ViT’s superior performance over other neural network models. The curves for ViT consistently outperformed those of ResNet50 and InceptionResNetv2 across various prediction score thresholds. These findings indicate that ViT exhibits superior performance compared to other models in terms of LVRR prediction for individual images.

### 3.4 Performance evaluation of LVRR prediction for individual patients

We applied our proposed method, TRAITER, to predict LVRR for individual patients based on dual-stained images of nuclei and DNA damage marker in cardiac tissue. Patients were categorized into LVRR-positive or LVRR-negative classes, and prediction labels for each patient were determined through a majority vote based on predicted labels of patches from that patient within the test set of the dual-stained image dataset. ResNet50, InceptionResNetv2, and ViT were compared, and the number of accurately predicted labels, along with the corresponding accuracy rates, were calculated (Table 3).

**Table 3.** Accuracy rate of LVRR prediction for individual patients from dual-stained images of nuclei and DNA damage marker.^a^

	ResNet50	InceptionResNetv2	Vision Transformer (ViT)
Accuracy rate	10/14	12/14	13/14
	(71.4)	(85.7)	(92.9)

a Number of correct predictions and accuracy rate for each model.

ResNet50 accurately identified 10 out of 14 LVRR cases, achieving an accuracy rate of 71.4%. InceptionResNetv2 demonstrated improved performance by correctly identifying 12 out of 14 LVRR cases, resulting in an accuracy rate of 85.7%. ViT exhibited the highest performance, accurately identifying 13 out of 14 LVRR cases, with the highest accuracy rate at 92.9%. ResNet50 displayed lower accuracy in distinguishing LVRR-negative patients, accurately identifying only four out of seven cases (Supplementary Table S1). In contrast, InceptionResNetv2 correctly identified six out of seven cases, encompassing both LVRR-positive and LVRR-negative patients, indicating enhanced accuracy (Supplementary Table S2). ViT demonstrated superior performance by accurately distinguishing six out of seven LVRR-negative patients and correctly identifying all seven LVRR-positive patients (Supplementary Table S3). These results underscore the superior performance of ViT compared to other models in predicting LVRR for individual patients.

## 4 Discussion

In this study, we demonstrated the usefulness of our TRAITER method for HF prediction and for LVRR prediction. Our model exhibited efficacy in predicting HF from images of nuclear morphology in cardiac tissue and demonstrated the feasibility of LVRR prediction for both individual images and individual patients through dual-stained images of nuclei and DNA damage marker. While there are previous works on the use of electrocardiogram waveforms for research on HF (Katsushika *et al.* 2021, Sawano *et al.* 2022), this study marks the first application of deep learning to predict HF from stained images of nuclear morphology and predict LVRR from dual-stained images of nuclei and DNA damage marker at the patient level.

A distinguishing feature of our deep learning model is the implementation of the Vision Transformer (ViT), which enhances prediction accuracy with image data. Unlike convolutional neural networks (CNNs), ViT learns correlations among segmented input images without relying on convolutional layers for feature extraction. This lack of convolutional bias enables ViT to comprehend images globally (Saha 2018, Dosovitskiy *et al.* 2021, Maurício *et al.* 2023). In the HF and LVRR prediction tasks, ViT consistently outperformed other neural network models, including CNN. Notably, the use of dual-stained images resulted in higher accuracy compared to the use of either images stained for nuclei alone or images stained for DNA damage marker alone. Given the reported associations between LVRR and myocardial DNA damage (Ko *et al.* 2019, Dai *et al.* 2024), and those between HF and altered nuclear morphology in cardiac tissue (Ross and Stroud 2021, Yamada *et al.* 2023), the combined use of both nuclei and DNA damage marker images proved synergistic, leading to improved prediction accuracy. While CNN excels at extracting essential local image features, it may overlook non-extracted image features. Dual-stained images, encompassing both nuclei and DNA damage marker across multiple cardiac tissues, may lose information when specific image portions are emphasized by the CNN model. In contrast, ViT, free from convolution bias, learns and predicts images from a global perspective, effectively incorporating information from multiple nuclei and DNA damage marker signals. These capabilities of the ViT model likely contributed to the achievement of the highest accuracy.

Accurate diagnosis and prognosis estimation are crucial in determining optimal diagnosis and treatment for individual patients in clinical practice. HF, characterized by diverse causes and symptoms, necessitates varied diagnostic and treatment approaches (Metra and Teerlink 2017). Patients with a poor prognosis may require heart transplantation, yet the shortage of donor hearts poses a significant challenge, limiting the number of patients who can undergo the heart transplantation and prolonging the wait time (Khush *et al.* 2015, Cameli *et al.* 2022). Estimating prognosis before treatment, alongside accurate diagnosis, enables the provision of appropriate and timely medical interventions. Medical practitioners can prioritize recommending ventricular assist devices or heart transplantation for patients with unfavorable prognosis. This study introduces new indicators for diagnosis and treatment decisions in clinical practice, contributing to precision medicine tailored to individual patients. The proposed method’s applicability extends to other diseases, using pathological image-derived elements as indicators for disease and prognosis prediction. Future efforts will involve investigating the dependence of prediction accuracy on the number of images used for learning and assessing the robustness of prediction accuracy across datasets captured in different facilities and under different conditions.

## Supplementary Material

btae610_Supplementary_Data
