# Supplementary material for: TRAITER: transformer-guided diagnosis and prognosis of heart failure using cell nuclear morphology and DNA damage marker
Source: Bioinformatics. 2024 Oct 16;40(11):btae610. doi: 10.1093/bioinformatics/btae610 (PMC11552630; doi:10.1093/bioinformatics/btae610)
Supplement: btae610_Supplementary_Data [file btae610_supplementary_data.docx]

Supplementary Information

**Transformer-guided diagnosis and prognosis of heart failure using cell nuclear morphology and DNA damage marker**

**Hiromu Hayashi^1^**, **Toshiyuki Ko^2,3^, Zhehao Dai^2^, Kanna Fujita^2,4^, Seitaro Nomura^2,5^*, Hiroki Kiyoshima^1^, Shinya Ishihara^1^, Momoko Hamano^1^*, Issei Komuro^2,5,6^*, Yoshihiro Yamanishi^1,7,^***

*** To whom correspondence should be addressed.**

^1^Department of Bioscience and Bioinformatics, Faculty of Computer Science and Systems Engineering, Kyushu Institute of Technology, 680-4 Kawazu, Iizuka 820-8502, Fukuoka, Japan

^2^Department of Cardiovascular Medicine, Graduate School of Medicine, The University of Tokyo, 7-3-1 Hongo, Bunkyo, Tokyo, 113-8655, Japan

^3^Department of Therapeutic Strategy for Heart Failure, Graduate School of Medicine, The University of Tokyo, 7-3-1 Hongo, Bunkyo, Tokyo, 113-8655, Japan

^4^Department of Computational Diagnostic Radiology and Preventive Medicine, Graduate School of Medicine, The University of Tokyo, 7-3-1 Hongo, Bunkyo, Tokyo, 113-8655, Japan

^5^Department of Frontier Cardiovascular Science, Graduate School of Medicine, The University of Tokyo, 7-3-1 Hongo, Bunkyo, Tokyo, 113-8655, Japan

^6^ International University of Health and Welafare, 4-1-26 Akasaka, Minato, Tokyo 107-8402, Japan

^7^ Department of Complex Systems Science, Graduate School of Informatics, Nagoya University, Chikusa, Nagoya, Aichi 464-8601, Japan.

This file includes

Supplementary Figure 1. Generation of cell nuclear morphology images of cardiac tissue to predict HF

Supplementary Figure 2. Generation of dual-stained images and patches to predict LVRR

Supplementary Figure 3. Vision Transformer (ViT) model architecture

Supplementary Figure 4. Comparative analysis of LVRR prediction impact among different image types

Supplementary Methods

Supplementary Discussion

**Supplementary Figures**


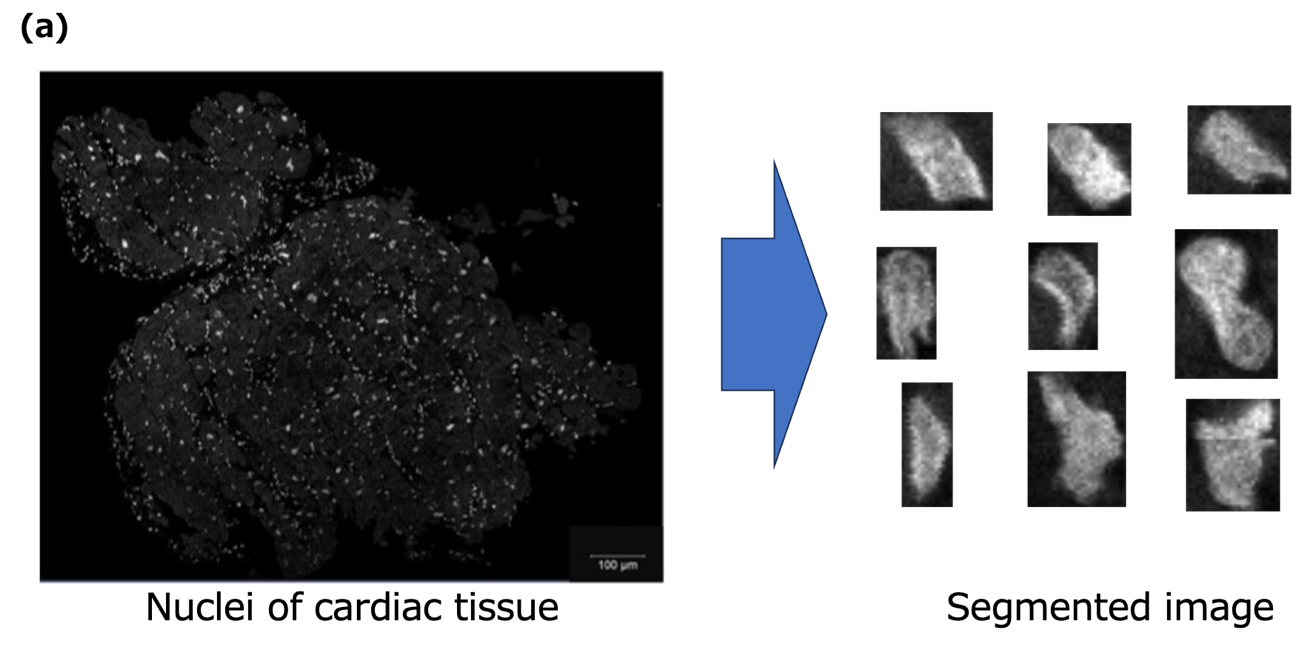


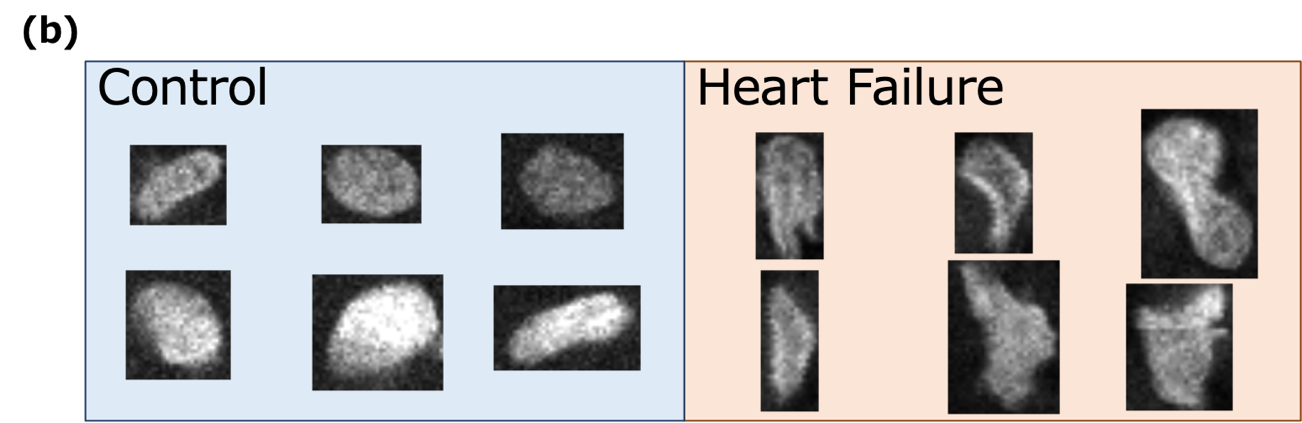

**Supplementary Fig 1. Generation of cell nuclear morphology images of cardiac tissue to predict HF.**

**(a)** Example of a high-resolution image of cardiac tissue stained for nuclei and segmentation for generation of nuclear morphology images. **(b)** Nuclear morphology images of control subjects and heart failure (HF) patients. **(c)** Number of nuclear morphology images segmented from each patient – blue bars for HF patients and red bars for control subjects.

**Supplementary Fig 2. Generation of dual-stained images and patches to predict LVRR.**

**(a)** Example of images stained for and for DNA damage marker. **(b)** Overlay of the dual-stained images. **(c)** Overview of segmentation from the dual-stained images.

**Supplementary Fig 3. Vision Transformer (ViT) model architecture.**

**(a)** Model architecture of the Vision Transformer (ViT) used in this study. We divided the input image into patches of fixed size. We then sent it to the sequence of vectors obtained by linear embedding and adding positional embedding. Classification was then performed using a multilayer perceptron. **(b)** Structure of transformer encoder. The transformer encoder consists of alternating layers of multi-head attentions and MLP blocks. Layer norm is applied before every block and the residual connections are applied after every block.

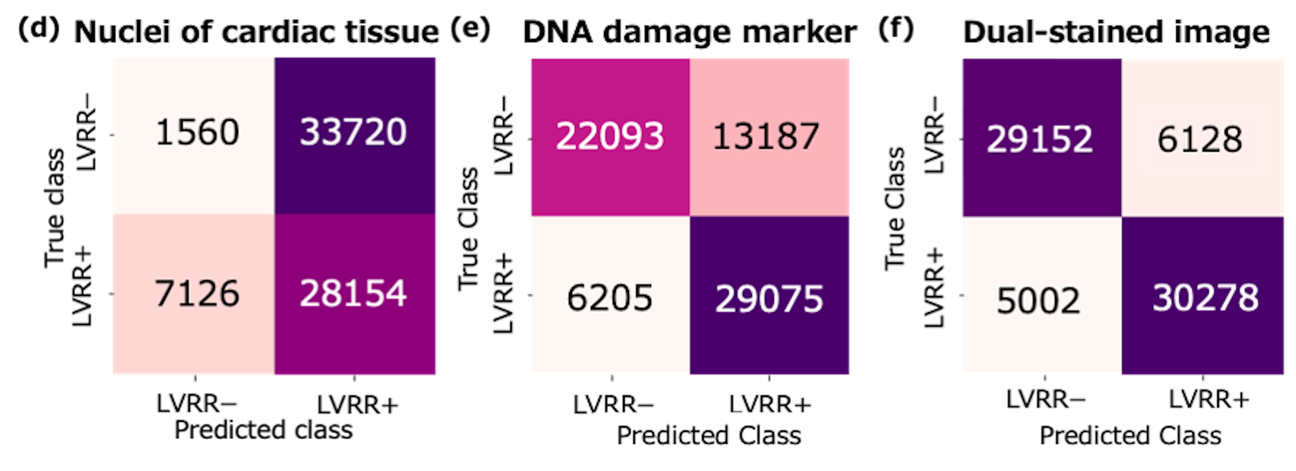


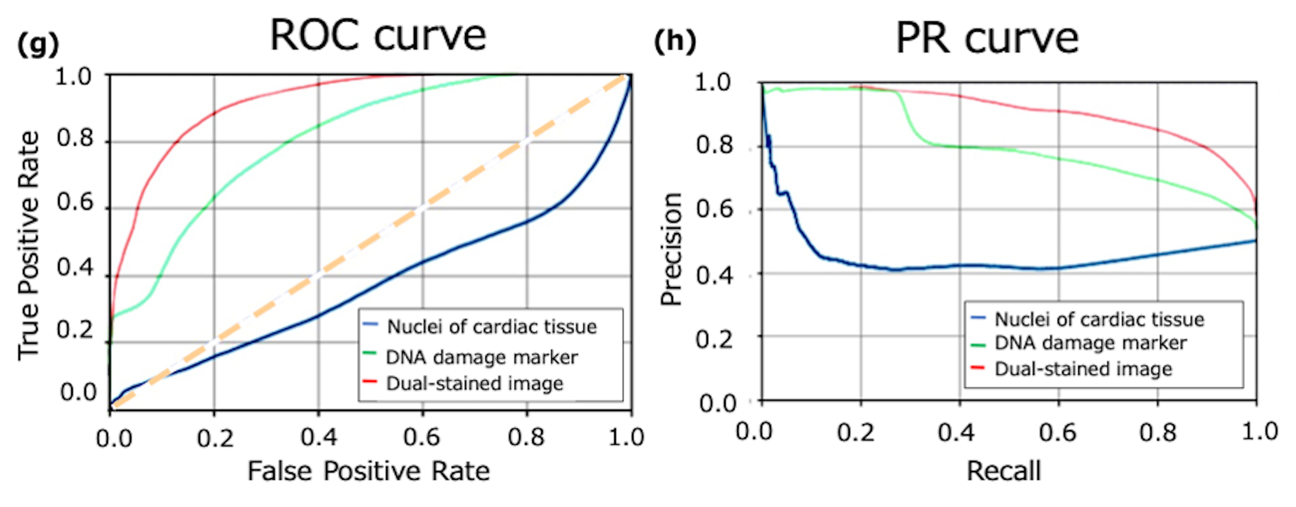


**Supplementary Fig 4. Comparative analysis of LVRR prediction impact among different image types.**

**(a)** Accuracy for each image type – blue bar for images stained for nuclei, green bar for images stained for DNA damage marker, and red bar for dual-stained images. **(b)** AUC scores for each image type – blue bar for images stained for nuclei, green bar for images stained for DNA damage marker, and red bar for dual-stained images. **(c)** AUPR scores for each image type – blue bar for images stained for nuclei, green bar for images stained for DNA damage marker, and red bar for dual-stained images. **(d)** Confusion matrix of images stained for nuclei, **(e)** Confusion matrix of images stained for DNA damage marker **(f)** Confusion matrix of the dual-stained images. **(g)** ROC curves for each image type—blue line for images stained for nuclei, green line for images stained for DNA damage marker, and red line for dual-stained images. **(h)** PR curves for each image type – blue line for images stained for nuclei, green line for images stained for DNA damage marker, and red line for dual-stained images.

**Supplementary Methods**

**Vision Transformer (ViT)**

Vision Transformer (ViT) is a neural network that applies Transformer to the image field. The input image data was resized to 224 x 224 pixels and divided into 196 patches of 16 x 16 pixels in size with no overlapping areas. Each patch was made in a one-dimensional manner by Linear Projection of Flattened Patches, and linear projection was performed to obtain an output, which is called Patch Embedding. The process of Linear Projection of Flattened Patches and Position Embedding is expressed by the following equation (1).

$$\begin{aligned} \boldsymbol{z}_{0}=\left[ \boldsymbol{x}_{class}; \boldsymbol{x}_{1}^{\boldsymbol{p}}\boldsymbol{E}; \boldsymbol{x}_{2}^{\boldsymbol{p}}\boldsymbol{E}\ldots; \boldsymbol{x}_{n}^{\boldsymbol{p}}\boldsymbol{E} \right]+\boldsymbol{E}_{pos}\#\left( 1 \right) \end{aligned}$$

where $\boldsymbol{z}_{0}$ is Patch Embedding, $\boldsymbol{x}_{class}$ is a class token, $\boldsymbol{x}^{\boldsymbol{p}}$ is a patch, ***E*** is a matrix for converting the patch to one dimension, and $\boldsymbol{E}_{pos}$ is location information. Note that $\boldsymbol{z}_{0}\in\boldsymbol{R}^{\left( n+1 \right)\times D}$, $\boldsymbol{x}_{class}$ is a D-dimensional vector, $\boldsymbol{x}^{p}\in\boldsymbol{R}^{1\times(P^{2}C)}$, $\boldsymbol{E}\in\boldsymbol{R}^{(P^{2}C)\times D}$, $\boldsymbol{E}_{\boldsymbol{pos}}\in\boldsymbol{R}^{(n+1)\times D}$. The Transformer encoder is then passed through 12 times; Attention is represented by Equation (2) and the Transformer multilayer perceptron (MLP) by Equation (3).

$$\begin{aligned} \boldsymbol{z}_{l}^{\boldsymbol{'}}=MSA\left( LN\left( \boldsymbol{z}_{l-1} \right) \right)+\boldsymbol{z}_{l-1}\#\left( 2 \right) \end{aligned}$$

$$\begin{aligned} \boldsymbol{z}_{l}=MLP\left( LN\left( \boldsymbol{z}_{l}^{'} \right) \right)+\boldsymbol{z}_{l}^{\boldsymbol{'}}\#\left( 3 \right) \end{aligned}$$

where **z** is the output of Patch Embedding or one previous Transformer, l is a number indicating how many times the Transformer Encoder is used, MSA stands for Multi-Head Self-Attention and is expressed by equations (4) and (5), and LN stands for Layer Normalization, which is one of the normalization methods that perform normalization for each layer of the same layer.

$$MultiHead\left( \boldsymbol{Q,K,V} \right)=Concat\left( \boldsymbol{hea}\boldsymbol{d}_{1}\boldsymbol{, \ldots, hea}\boldsymbol{d}_{h} \right)\boldsymbol{W}^{O}$$

$$\begin{aligned} where \boldsymbol{hea}\boldsymbol{d}_{i}=Attention\left( \boldsymbol{Q}\boldsymbol{W}_{i}^{\boldsymbol{Q}}\boldsymbol{, K}\boldsymbol{W}_{i}^{\boldsymbol{K}}\boldsymbol{, V}\boldsymbol{W}_{i}^{\boldsymbol{V}} \right)\#\left( 4 \right) \end{aligned}$$

$$\begin{aligned} Attention\left( \boldsymbol{Q},\boldsymbol{K},\boldsymbol{V} \right)=softmax\left( \frac{\boldsymbol{Q}\boldsymbol{K}^{T}}{\sqrt{d_{k}}} \right)\boldsymbol{V} \#\left( 5 \right) \end{aligned}$$

Here, ***Q*** is the matrix of Query that indicates what we want to search, and **K** and **V** are the matrices of Key and Value, which are dictionary objects corresponding to each other. Note that $\boldsymbol{Q}\in\boldsymbol{R}^{d_{w}\times d_{k}}$, $\boldsymbol{K}\in\boldsymbol{R}^{d_{w}\times d_{k}}$, and $\boldsymbol{V}\in\boldsymbol{R}^{d_{w}\times d_{v}}\boldsymbol{,}\boldsymbol{W}^{\boldsymbol{O}}\in\boldsymbol{R}^{{hd}_{v}\times d_{model}}\boldsymbol{,}\boldsymbol{W}^{\boldsymbol{Q}}\in\boldsymbol{R}^{{hd}_{model}\times d_{k}}\boldsymbol{,}\boldsymbol{W}^{\boldsymbol{K}}\in\boldsymbol{R}^{d_{model}\times d_{k}}\boldsymbol{,}\boldsymbol{W}^{\boldsymbol{V}}\in\boldsymbol{R}^{d_{model}\times d_{v}}$. Also, softmax is a function that converts values so that the sum of output values is 1. Note that $\boldsymbol{z}\in\boldsymbol{R}^{\left( n+1 \right)\times D}$. Then, after passing through the multilayer perceptron, the output was classified into two classes, and the values were input to the sigmoid function as 0~1 values.

**Supplementary Discussion**

**The impact of using multiple control individuals instead of just one.**

The image datasets for heart failure (HF) prediction and left ventricular reverse remodeling (LVRR) prediction in this study comprised stained images obtained from different biopsy samples. The dataset for HF prediction is particularly limited, as myocardial biopsies from control individuals are sourced from transplanted myocardium. Only three such samples were available for this study. In the cross-validation experiment, only one sample was allocated to the test set. However, the data expansion techniques introduced in this study enabled effective discrimination analysis using deep learning.

It is important to note that the images used in this study are not publicly available data from existing databases; they are original data obtained through biopsies and stained image preparation directly from patients at the University of Tokyo Hospital. This process required significant human effort and the cooperation of patients. We would like to ask for your understanding that conducting biopsies is feasible in only a limited number of patients. If biopsies of transplanted myocardium can be obtained from a larger number of patients in the future, we intend to explore the impact of increasing the number of images on prediction accuracy.

**The image quality and features in Supplementary Figure 1.**

The images used in this analysis were taken under conditions that mimic actual clinical settings. Each image captured the entire myocardial biopsy section within a single field of view. High magnification was not used to capture individual nuclei in detail, as this would require advanced skills and could be challenging for physicians in a clinical setting. Supplementary Figures 1a and 1b were initially low-resolution images; therefore, they have been redrawn and replaced with higher-quality versions.

As noted, distinguishing DNA characteristics in these images can be challenging for the human eye. The image of the HF group in Supplementary Figure 1b might appear to show a slightly higher background and variation in staining intensity. However, this is merely an example derived from a few images, and there may not be a significant difference in staining intensity between the control and HF groups across all images. We believe that the deep learning model effectively extracts and differentiates key features of nuclear morphology, such as shape, size, and circularity, from the images.

**The feature extraction obtained from the nuclear morphology images by deep learning.**

We believe the deep learning model effectively extracts and distinguishes key features of nuclear morphology, such as shape, size, and circularity, from the images. Mutations in LMNA, which encodes lamin A/C—a major component of the nuclear lamina that causes abnormalities in nuclear morphology in myocardial tissue—are a primary cause of dilated cardiomyopathy (DCM) (Yamada et al., Science Advances, 9 (15), eade7047 (2023)). Several studies have reported an association between abnormalities in nuclear morphology in myocardial tissue and heart failure (Mounkes et al., Hum. Mol. Genet. 14, 2167-2180 (2005); Chatzifrangkeskou et al., Hum. Mol. Genet. 27, 3060-3078 (2018); Vignier et al., Cell Rep. 36, 109601 (2021)). Although it is challenging to detect significant morphological differences between the control and heart failure groups in Supplementary Figure 1c with the human eye, it is noteworthy that the deep learning model can identify subtle nuclear morphological features and accurately distinguish the presence or absence of heart failure.

**The comparison between image stack projections and individual optical sections.**

Comparing the results of analyses using images with different individual optical sections can provide meaningful insights. However, it is challenging to prepare new images by altering the z-axis while maintaining identical myocardial biopsy tissue sections. Given that the same nuclei are photographed in images with varying z-axis orientations, we believe that the discrimination results would not significantly change.

**On the clinical practicality of using cardiac biopsies for patient risk assessment.**

Indeed, endomyocardial biopsy is an invasive procedure, and its use should be carefully considered in clinical settings. However, biopsy is still recommended for diagnosing patients with suspected cardiomyopathy (ESC Guidelines, Eur Heart J 2023; 44: 3503-3626). Additionally, previous studies have shown that evaluating biopsy specimens can improve risk stratification for future events. Thus, diagnosing heart failure and predicting LVRR through deep learning analysis of biopsy specimen images provides a novel and accurate method for risk stratification. This approach also maximizes the utility of valuable biopsy specimens collected through invasive means.
